# Our Vienna Letter

**Published:** 1890-05

**Authors:** Everard H. Richardson

**Affiliations:** Vienna, Austria


### OUR VIENNA LETTER.

VIENNA, AUSTRIA, March 28th, 1890.

For medical observation the Austrian capital—Vienna—stands deservedly high. Aside from the celebrity of her teachers in the several branches of medicine and surgery, Vienna's chief advantage is the concentration of her clinical material—drawn from a population of a million and a quarter people—into one large general Hospital practically under one roof. The several wings of this colossal building are connected by a network of courts and corridors, the aggregated capacity of which affords three (3000) thousand beds. Here more than twenty-six thousand patients are treated annually From this number twenty-seven (2700) hundred *post mortem* examinations are made during the year. With a corps of one (150) hundred and fifty teachers and assistants, every variety of disease may be studied in the wards of the Hospital. At the *polyclinic* (also connected with the Hospital) upwards of forty (40,000) thousand patients are treated per annum. The lying in department of the hospital contains six (600) hundred beds, in which ten (10,000) thousand births occur a year. Carl Braun, although he is seventy years old, is still connected with this branch of the hospital, and does gynecological work in the amphitheatre before a class of appreciative students. Above his operating table two antiseptic solutions are observed. Thymol 1: 1000. Sublimate 1: 4000.

Upon entering the maternity department for confinement a woman is immediately required to take a general warm bath. She is afterwards examined under antiseptic restrictions. The vagina is irrigated before, during and after accouchment with a sublimate solution. After confinement she is dressed and treated precisely as after the performance of a capital operation, and not as though she had passed through a physiological process. They claim that by these means puerperal and septic troubles are obviated and mortality reduced to a minimum. The truth is in the entire workings of this hospital the principal of *antisepsis* as first enunciated and promulgated by Mr. Lister are recognized and most scrupulously enforced from alpha to omega. No operation is performed without paying tribute to the greatest benefactor of modern times, Sir Joseph Lister.

The American medical student is found here in goodly numbers. A limited number of English students also annually congregate here. The facilities for *post graduate* study in the laboratory, upon the operating and *post mortem* tables, are most excellent. Special private practical courses in every branch of medicine and surgery are given by thorougly qualified men continuously throughout the year. Some of these courses are given in both the English and German language. But as a matter of fact, the student who does not understand the German language labors under a great disadvantage. Patients here are treated as *material*—clay in the potter's hand—to subserve the purposes of science, in teaching and demonstration either upon the operating or *post-mortem* table. In this country "liberty is not kept on tap," and the patients don't seem to mind it. The hospital surgeon does not grope in the dark long. If he is in doubt, he gives the patient the benefit of the doubt by boldly cutting down, and doing an explanotory operation; and their results are marvelously brilliant. An immense amount of knowledge is the outcome of such methods, and ultimately I opine human life in the aggregate is increased.

To the medical mind seeking to add to its stock of information, Vienna offers a capital field, barring *therapeutics*, which seems to be deemed a question of minor importance. But for the study of diagnosis and pathology, with the elementary branches of both medicine and surgery, the opportunities for the acquisition of practical and theoretic knowledge are all that could be desired. Among the names of the living that have contributed as teachers and *Savants* to Vienna renown as a medical centre, may be mentioned as conspicuous, Billroth, Carl and Gustave Braun, Northragee, Newman, Dillet, Theodore Maynert, Laposi Pollitzer, Kundrat, Kraft, Ebbing. Scraeder, Benekikt. Albert, Rotkitanky, Hebra, and a host of others. But perhaps the name of Billroth shines with greater lustre than that of any other whose name is associated with medicine on the continent of Europe, and the greatest interest and attraction is

PROF. BILLROTH'S SURGICAL CLINIC.

I am told on good authority that he is a native of Switzerland. His height is about five ft. nine inches, and I should judge his weight to be close to 175 lbs. His complexion is light, with dark gray eyes, and at the age of sixty-two, of course he is gray. His shoulders are slightly "stooped," though he is well preserved, and apparently has yet many years of useful work ahead of him. Seemingly his temperament is phlegmatic, 'tho while lecturing or talking he is rarely still. He is not considered a good lecturer; talks slowly, deliberately and low to indistinctness. Though rather reticent and reserved in manner, his face is kindly and genial, and but feebly portraying the real strength and power of the man back of it. He is plain and simple in demeanor and garb, suave and courteous to all with whom he comes in contact. So much for the personality of the man. But his methods as a surgeon and operator more nearly concern us.

The amphitheater in which he operates daily from 10 to 12 o'clock, except Saturdays, is the embodiment and personification of plainness and hardness (especial emphasis to be placed upon latter), the seating capacity of which is only about two hundred persons. He has eight well trained assistants whom he directs and controls apparently without effort or friction, each one doing his assigned duty with the precision and alacrity, of a well drilled soldier. Before he enters the operating room everything is in readiness. Instruments are immersed in the standard solution of carbolic acid; fountains are filled with two solutions sublimate—one a strength of 1-1000, the other 1-2000; dressings and appliances are in order; a special case of instruments marked "Phlegmon" is conspicuous. Without ado the distinguished surgeon enters, and usually finds a patient under anesthesia, when he begins at once his operative work. Possibly his clinic is the richest in the world, there being no limit to the number of "nasty" cases presented to him for operative interference. Before beginning an operation he not infrequently remarks that he does not know the character of the malady before him, but that he proposes to make an exploratory operation, with the object of ascertaining, when he deals with it as the exigencies of the case demand. One does not observe the surgeon and his methods long before he concludes, "the man is a marvel as a surgeon, and richly deserves all the lustre and matchless splendor that clusters about his name." The utmost simplicity, intrepidity without rashness, efficiency without hurry, consummate mastery of his subject, coupled with perfect coolness, characterize him as an operator. Without display or ostentation he is in any emergency supreme master of the situation, and is artistically deft and expert in the execution of his work. He is a most cordial believer in antiseptic surgery as taught by Lister. Next in importance to irrigation with sublimate solution, he prizes filling all wounds with the following solution of iodoform:

| | | |
|---|---|---|
| Iodoform, | grams | 50. |
| Glycerin | " | 100. |
| Alcohol | " | 700. |
| Ad alcohol | | 2. s. |
| Ft. Litres | | 10. |

To further illustrate his methods of wound treatment, a few cases will be given as occurring in his clinic.

CASE 1.—Compound commutated fracture of elbow joint. Posterior opening enlarged and fragments of bone removed, joint irrigated with sublimate solution 1-2000, filled with solution iodoform and wound closed with continuous silk suture, without use of a drainage tube, when the joint was immobilized by application of plaster paris dressing.

CASE 2.—Extensive abscess, involving all the tissues of thigh. Free incision made, irrigation and drainage established, supplemented by roller bandage. *Post mortem* demonstrated that this case at times was suffering from acute nephritis.

CASE 3.—Chronic pyothorax of left lung. Five ribs were resected in front, both lobes of lung being made visible; pleural cavity thoroughly irrigated with sublimate and iodoform solutions, completed by stuffing cavity with iodoform gauze.

CASE 4.—Scrofulous knee joint having been previously laid wide open was irrigated with sublimate and iodoform solutions, sutured with silk ligature, and plaster paris dressing applied with limb in semifixed position, without drainage tube.

CASE 5.—Examined patient and pronounced it to be an abscess of abdominal cavity, but said that whether it had its origin in kidney or caecum he did not know. Made incision in right side as for colotomy. Large amount of extremely foetid pus evacuated, counter opening made in front and a glass drainage tube left in this opening. The character of the abscess proved to be a perityphlitis one, and after irrigation the cavity was filled with gauze and the usual antiseptic dressings placed over this.

CASE 6.—Large ovarian cyst. By careful examination he ascertained that extensive adhesions existed, and that the intestines were out of harm's way, with one sweep of the bistory he cut through abdominal walls, through cyst and evacuated contents without canula. Without enlarging opening he introduced hand and broke up adhesions capable of being thus dealt with. Those that he could not break up with fingers he ligated and burned off with cautery. Ligated pedicle with silk ligature and burnt off with cautery and closed abdomen without drainage tubes.

These cases, taken from my note book just as they occurred at Billaeth's clinic, are sufficient to give his methods of treatment of wounds as now practiced by him. I will briefly mention that chloroform largely dominates in the anaesthetic used by him, which is

| | | |
|---|---|---|
| Chloroform | - | 200 parts. |
| Alcohol | | |
| Ether sul. *av.* | - | 60 parts. |

M.

Given in an inhaler very simple in construction, and, I should say, excludes less than 60 per cent. of ordinary atmosphere. Is it safer or as *safe* as ether alone? I think not. In England and the continental hospitals chloroform is given much oftener than ether. The Hyderabad chloroform commission tells us, in their report found in the February issue of London Lancet, that "it is impossible to produce (primary) syncope in dogs from chlororm," and further that "chloroform may be given in any case requiring an operation with perfect ease and absolute safety." With the *clinician* such laboratory work performed upon the lower animals will be received *cum grano salis.*

One exception upon the human subject explodes the theory set up by experiment on a thousand dogs. During one week in Europe I observed three cases where in beriam of the patient coupled with artificial respiration was deemed necessary to save the patients from impending death. Unquestionably the administration of chloroform demands more skill and greater vigilance than ether.

The number of medical students who attend Albert's surgical clinic is even greater than those who frequent Billroth's, but of his work and that of others I shall not at present speak. The length of this letter admonishes me not to further trespass upon your valuable space, nor to tax too severely the patience of your numerous readers.

Very much more might be said of Vienna as a medical emporium, but for cogent reasons I shall desist by suggesting that should any of your readers contemplate a visit here they write before coming to the Anglo-American Medical Association Landesgerichts, Strasse No. 12, Vienna, for information.

Very respectfully, EVERARD H. RICHARDSON.

